# Preparation of Pd/C by Atmospheric-Pressure Ethanol Cold Plasma and Its Preparation Mechanism

**DOI:** 10.3390/nano9101437

**Published:** 2019-10-10

**Authors:** Zhuang Li, Jingsen Zhang, Hongyang Wang, Zhihui Li, Xiuling Zhang, Lanbo Di

**Affiliations:** College of Physical Science and Technology, Dalian University, Dalian 116622, China; lizhuang668@sina.com (Z.L.); 18742089936@163.com (J.Z.); hywang68@sina.com (H.W.); lee6062@163.com (Z.L.)

**Keywords:** ethanol cold plasma, hydrogen cold plasma, Pd/C, CO oxidation, preparation mechanism

## Abstract

Treatment with atmospheric-pressure (AP) hydrogen cold plasma is an effective method for preparing highly active supported metal catalytic materials. However, this technique typically uses H_2_ as working gas, which is explosive and difficult to transport. This study proposes the use of PdCl_2_ as a Pd precursor and activated carbon as the support to fabricate Pd/C catalytic materials (Pd/C-EP-Ar) by using ethanol—which is renewable, easily stored, and safe—combined with AP cold plasma (AP ethanol cold plasma) followed by calcination in Ar gas at 550 °C for 2 h. Both Pd/C-EP and Pd/C-HP fabricated using AP ethanol and hydrogen cold plasma (without calcination in Ar gas) respectively, exhibit low CO oxidation reactivity. The activity of Pd/C-EP is lower than Pd/C-HP, which is mainly ascribed to the carbon layer formed by ethanol decomposition during plasma treatment. However, the 100% CO conversion temperature (*T*_100_) of Pd/C-EP-Ar is 140 °C, which is similar to that of Pd/C-HP-Ar fabricated using AP hydrogen cold plasma (calcined in Ar gas at 550 °C for 2 h). The characterization results of X-ray diffraction, X-ray photoelectron spectroscopy, and transmission electron microscopy indicated that the carbon layer formed by ethanol decomposition enhanced the interaction of metal nanoparticles to the support, and a high Pd/C atomic ratio was obtained. This was beneficial to the high CO oxidation performance. This work provides a safe method for synthesizing high-performance Pd/C catalytic materials avoiding the use of H_2_, which is explosive and difficult to transport.

## 1. Introduction

Supported Pd catalytic materials are widely used in organic catalytic hydrogenation, CO oxidation, automobile exhaust treatment, and the Suzuki reaction, among others [1,2,3,4,5,6]. Thus, they have drawn interest for their potential use in the fabrication of supported Pd catalytic materials with high metal utilization, high activity, and long service life by using efficient, safe, simple, and low-cost methods. Although traditional impregnation, ion exchange, sol-gel, and vapor deposition methods can be applied to prepare highly active Pd catalytic materials, the fabrication process is generally complex and difficult to control [7,8,9,10]. Atmospheric-pressure (AP) cold plasma treatment is a fast and effective method for preparing metal catalytic materials with small metal nanoparticles and high activity. Owing to these properties, the aforementioned approach has been broadly applied in the fabrication of Pd catalytic materials [11,12,13,14].

As a non-equilibrium plasma, H_2_ or NH_3_, CH_4_ has been added into the working gas in AP cold plasma due to the frequency of collision between its electrons and heavy particles, leading to inadequate electron temperature for direct metal ion reduction [15,16,17,18]. Metal ions on the support were reduced to their metallic states by the generated active hydrogen species (H*, H_2_*, etc.) in AP cold plasma. However, the aforementioned gases are explosive and are difficult to store and transport. Thus, the development of a relatively safe AP cold plasma for the preparation of supported metal catalytic materials bears research significance and exhibits application potential.

Ethanol, a hydrogen-rich renewable resource, can be mixed with water in any proportion and shows reducibility. Research on reforming hydrogen production from ethanol has recently gained interest [19,20,21]. Plasma technology has been used to reform hydrogen production from ethanol. Wang et al. used microwave plasma (2.45 GHz) to produce hydrogen from low carbon alcohol (methanol and ethanol) solutions and evaluated the effect of microwave power input and alcohol concentration on hydrogen production. Results indicated that not only hydrogen but carbon species (such as acetylene, CO and CO_2_) as well are produced during microwave discharge [22]. Wu et al. reported on the synthesis of a Ni/γ-Al_2_O_3_ catalyst by dielectric barrier discharge, which increased the conversion rate of ethanol to hydrogen by nearly 30%, meanwhile, the carbon balance was 97% [23].

In a previous study, metal (Au, Pd, Ag, Pt) ions loaded on Deggusa P25 (commercial titania) were successfully reduced by combining AP cold plasma with ethanol instead of H_2_, and Pd/P25 catalytic materials with high activity and small Pd nanoparticles were prepared by AP ethanol cold plasma treatment. The performance was better than that of the catalytic materials prepared using AP hydrogen cold plasma [24,25]. The structure, specific surface area, and other properties of supports influence the catalytic materials, thus, supports play an important role in these materials. Activated carbon has been widely used as a support because of its high-specific surface area (500–1700 m^2^·g^−1^) and rich oxygen-containing functional groups located on the surface. In addition, activated carbon is cheap and conducive to the recovery of precious metals. In this study, activated carbon was selected as the support, and AP ethanol cold plasma was used in the fabrication of Pd/C catalytic materials.

In this study, Pd/C catalytic materials were successfully prepared using AP ethanol cold plasma. The results of transmission electron microscopy (TEM), X-ray photoelectron spectroscopy (XPS), X-ray diffraction (XRD), and CO oxidation reaction show that the CO oxidation activity of Pd/C-EP-Ar prepared using AP ethanol cold plasma is similar to that of Pd/C-HP-Ar prepared using AP hydrogen cold plasma. A carbon protective layer can be formed during AP ethanol cold plasma preparation, which can effectively protect the Pd nanoparticles.

## 2. Experimental

### 2.1. Catalysts Preparation

Activated carbon (40–60 mesh, Beijing Guanghua Timber Mill., Beijing, China) was used as the support, and the aqueous solution of PdCl_2_ (AR, 99%, Tianjin Kemio Chemical Reagent Co., Ltd., Tianjin, China) was used as the Pd source. Prior to the fabrication of Pd/C catalytic materials, the activated carbon and 30 wt% HNO_3_ were mixed in a 250 mL conical flask and then stored in a constant-temperature water bath at 85 °C for 5 h. The mixture was washed with deionized water to neutral pH and then dried at 120 °C for 2 h for subsequent use [26,27]. The Pd/C (theoretical load of Pd = 5 wt%) precursor was prepared using the treated activated carbon impregnated in a PdCl_2_ solution for 12 h and then dried at 120 °C for 2 h. The Pd/C precursor was ultimately treated with AP ethanol cold plasma to prepare Pd/C catalytic materials.

**Figure 1:**
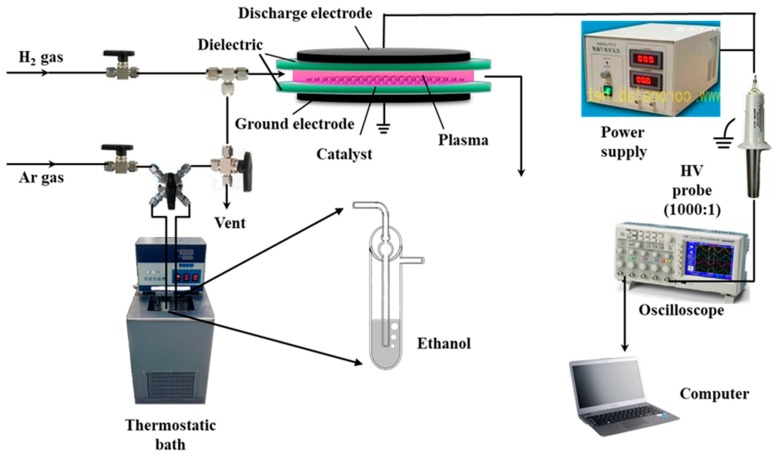
Schematic of atmospheric-pressure ethanol cold plasma treatment for the preparation of Pd/C catalytic materials.

The schematic of the Pd/C catalytic materials prepared using the AP ethanol cold plasma experimental device is presented in Figure 1. Plasma discharge was powered by a low-temperature plasma power (CTP-2000 K, Nanjing Suman Electronic Co. Ltd., Nanjing, China) at a 36 kV peak-to-peak discharge voltage of sinusoidal alternating current and a frequency of 13.4 kHz. A plate-to-plate type quartz reactor was placed between two parallel electrodes with a diameter of 50 mm and a discharge gap of 4 mm. Ethanol in a bubbler reactor in a 25 °C thermostatic bath was carried by 100 standard cubic centimeters per minute (SCCM) of Ar gas as the working gas. The partial pressure of ethanol was approximately 7.99 kPa, calculated using the saturated vapor pressure of ethanol. Treatment with AP ethanol cold plasma was conducted five times (at a discharge interval of 10 min) for 2 min each treatment. The Pd/C precursor treated with AP ethanol cold plasma is denoted as Pd/C-EP. For comparison, a Pd/C-HP sample was also prepared using AP hydrogen cold plasma with a mixture of Ar and H_2_ at 50 SCCM each, the mixture was used as the working gas. After calcination in 100 SCCM of Ar gas at 550 °C for 2 h, Pd/C-EP and Pd/C-HP are denoted as Pd/C-EP-Ar and Pd/C-HP-Ar, respectively.

### 2.2. Catalysts Characterization and Activity Test

The samples were characterized by XRD (DX-2700, Dandong Haoyuan Instrument Co., Ltd., Dandong, China) with graphite-monochromatized Cu Kα radiation (λ = 1.54178 Å) under the following conditions: Voltage, 40 kV; current, 30 mA; scan range, 5° to 90°. The chemical compositions and binding energies of the samples were investigated by XPS (ESCALAN-250, Thermo VG, Waltham, MA, USA) with a monochromatized Al Kα (1486.6 eV) X-ray source. All binding energies were corrected with C1s orbital binding energies at 284.6 eV. The morphology of the sample was investigated by TEM (HT7700, Hitachi Company, Tokyo, Japan), and the average size of the Pd nanoparticles was determined by selecting at least 200 nanoparticles. The specific surface area, pore volume, and pore size of the materials were measured using a NOVA 2200e gas sorption analyzer (Quantachrome Company, Boynton Beach, FL, USA) via nitrogen adsorption and desorption. CO oxidation was selected as a probe reaction to evaluate the activity of the prepared Pd/C catalytic materials in a quartz tube (inner diameter: 4 mm) placed in a temperature-programmed furnace. Detailed test conditions can be found in previous work [26].

## 3. Results and Discussion

**Figure 2:**
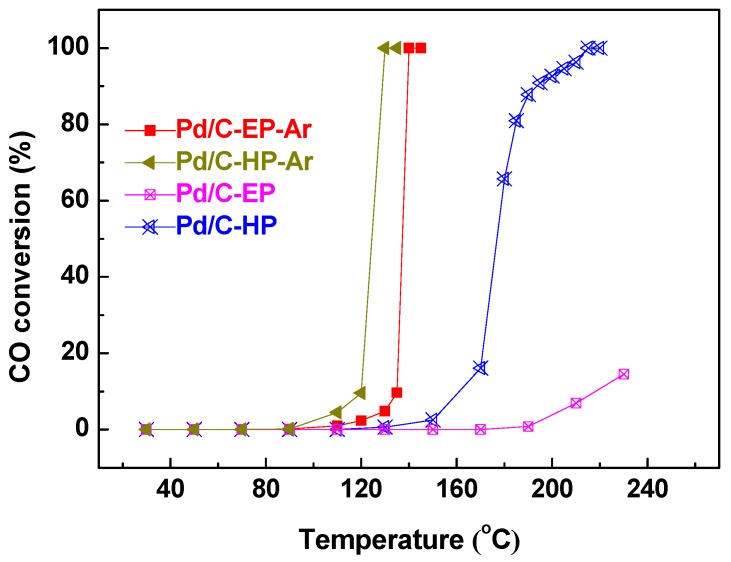
Catalytic activity of the catalytic materials Pd/C-EP-Ar, Pd/C-HP-Ar, Pd/C-EP, and Pd/C-HP for CO oxidation.

Figure 2 presents the CO oxidation reactivity of the Pd/C samples prepared by AP ethanol and hydrogen cold plasma. The 100% CO conversion temperature (*T*_100_) of Pd/C-EP-Ar, Pd/C-HP-Ar, and Pd/C-HP were 140 °C, 130 °C, and 215 °C, respectively. Notably, no CO oxidation activity over Pd/C-EP was observed even at 200 °C, and the conversion rate of CO at 230 °C was only 14.5%. The significantly lower CO oxidation reactivity over Pd/C-EP than that over Pd/C-HP is mainly attributed to the carbon layer formed by ethanol decomposition during plasma treatment, which will be discussed later. The results show that the *T*_100_ of Pd/C-EP-Ar is similar to the *T*_100_ of Pd/C-HP-Ar, indicating that AP ethanol cold plasma can achieve the same effect and ability as that of AP hydrogen cold plasma in the preparation of Pd/C catalytic materials, and its advantage is that the use of explosive H_2_ gas is prevented. In this work, the theoretical load of Pd is 5 wt%. The *T*_100_ of Pd/C-EP-Ar (140 °C) is close to the previously reported 6 wt% Pd/graphene (127 °C) fabricated by the thermal reduction method [3].

**Figure 3:**
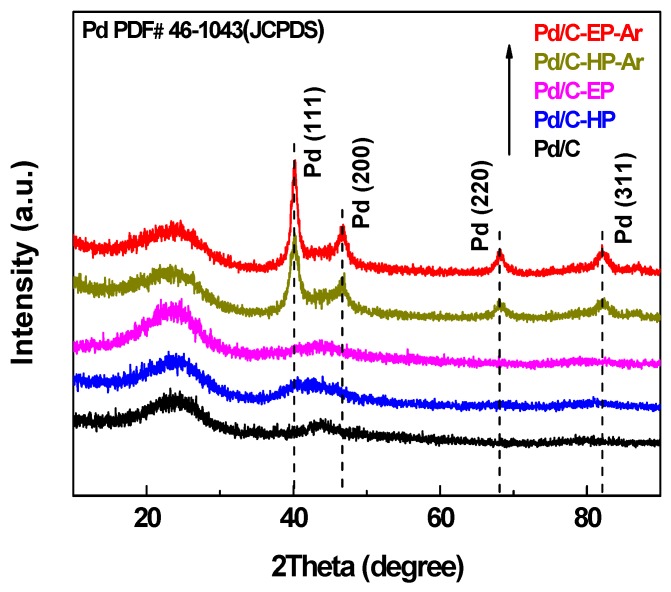
X-ray diffraction (XRD) patterns of Pd/C-EP-Ar, Pd/C-HP-Ar, Pd/C-EP, Pd/C-HP, and Pd/C.

The XRD patterns of the Pd/C-EP, Pd/C-EP-Ar, Pd/C-HP, Pd/C-HP-Ar, and Pd/C precursor are illustrated in Figure 3. The broad diffraction peaks at ca. 23°, 44° and 80° of all samples corresponded to the (002), (100) and (111) planes of graphitic carbon structure of the support [28]. These peaks are weak and broad, revealing a poor crystallite graphitic structure. Meanwhile, the characteristic diffraction peaks of Pd/C-EP-Ar and Pd/C-HP-Ar at 40.1°, 46.6°, 68.1°, and 82.4° corresponded to the (111), (200), (220), and (311) planes of metal Pd (JCPDS#46-1043), respectively. However, the Pd peaks for Pd/C-EP and Pd/C-HP were weaker than those of Pd/C-EP-Ar and Pd/C-HP-Ar, only the Pd peak at 40.1° was observed. The diffraction peak of Pd/C-EP at 25.0° was stronger than that of the support and other samples. This difference may be attributed to the carbon species formed on the material surface by ethanol decomposition during plasma discharge. Pd in Pd/C-EP-Ar and Pd/C-HP-Ar showed strong and sharp peaks, indicating that the nanoparticle size of Pd was relatively large owing to the agglomeration of Pd nanoparticles. Such agglomeration resulted from calcination at high temperatures in Ar gas.

**Figure 4:**
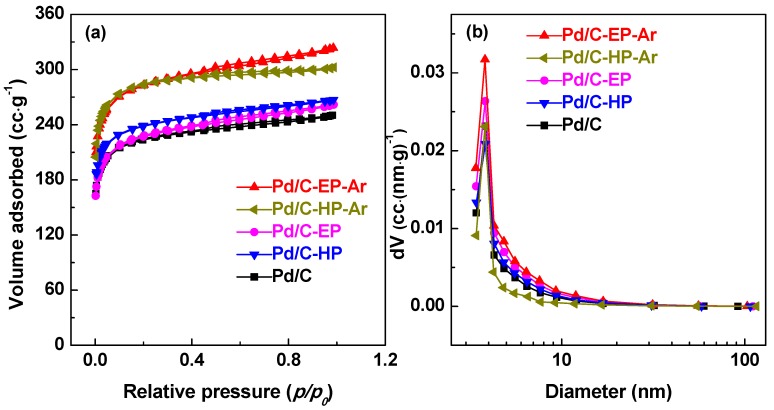
(**a**) N_2_ adsorption–desorption isotherms and (**b**) pore size distributions of Pd/C-EP-Ar, Pd/C-HP-Ar, Pd/C-EP, Pd/C-HP, and Pd/C.

To evaluate the effect of plasma treatment on the pore structure of the Pd/C catalytic materials, the N_2_ adsorption and desorption and pore size distribution of all samples were observed (Figure 4). The specific surface area, pore diameter, and pore volume of the Pd/C samples are summarized in Table 1. The Pd/C catalytic materials in descending order of specific surface area were Pd/C-EP-Ar > Pd/C-HP-Ar > Pd/C-HP > Pd/C-EP > Pd/C with the corresponding specific surface areas of 874, 871, 736, 703, and 690 m^2^·g^−1^, respectively (Figure 4a & Table 1). Correspondingly, the pore volumes of the Pd/C-EP-Ar and Pd/C-HP-Ar samples increased to 0.50 and 0.47 cm^3^·g^−1^, respectively. Compared to Pd/C-HP and Pd/C-EP, the specific surface area and pore volume of the Pd/C-EP-Ar and Pd/C-HP-Ar samples were increased, which may be mainly ascribed to the removal of the unstable carbon species in the presence of active Pd species during calcination.

**Figure 5:**
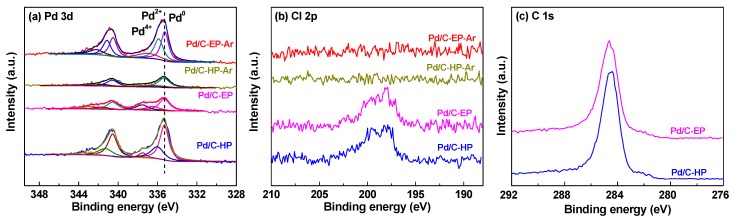
X-ray photoelectron spectroscopy (XPS) spectra of (**a**) Pd 3d and (**b**) Cl 2p in Pd/C-EP-Ar, Pd/C-HP-Ar, Pd/C-EP and Pd/C-HP, as well as (**c**) C1s XPS spectra in Pd/C-EP and Pd/C-HP.

XPS analysis was conducted to further investigate the valence states of the Pd/C catalytic materials, and the XPS spectra of Pd3d, Cl2p and C1s were measured (Figure 5). The peaks of Pd3d_5/2_ in all samples can be divided into three peaks at 335.3, 335.9, and 337.5 eV, which correspond to Pd^0^, Pd^2+^, and Pd^4+^, respectively [14,29]. The contents of the different valence states of Pd in the Pd/C catalytic materials were calculated based on the XPS spectra of Pd 3d (Table 2). The Pd/C atomic ratios in Pd/C-HP (0.026) was higher than that in Pd/C-HP-Ar (0.010) prepared by calcination. This difference was attributed to the active hydrogen species and high-energy electrons induced by AP hydrogen cold plasma treatment. Owing to these species, the Pd ions in the support channel were reduced and transferred to the surface, resulting in higher content of surface Pd components in Pd/C-HP [30]. In addition, there is a strong interaction between the Pd species and oxygen-containing functional groups on the surface of the support. Therefore, the Pd nanoparticles agglomerated and migrated back to the pore channels during calcination, resulting in a decrease in surface Pd in Pd/C-HP-Ar. Notably, a lower Pd/C atomic ratio in Pd/C-EP (0.013) was observed due to the protection of the carbon layer formed by ethanol decomposition during plasma treatment. However, it was increased to 0.023 for Pd/C-EP-Ar after calcination. The reason may be attributed to the destruction of the carbon layer at a high temperature, which led to the exposure of more Pd species than the as-prepared Pd/C-EP catalytic material. This is consistent with the XRD results, which indicated that the broad peak corresponding to carbon species in Pd/C-EP-Ar was obviously decreased compared to that for Pd/C-EP.

In Figure 5b, the characteristic peak of Cl 2p is observed in the range of 190 eV to 210 eV for both Pd/C-EP and Pd/C-HP, meanwhile, no such peak is found in Pd/C-EP-Ar and Pd/C-HP-Ar, which may be attributed to the removal of chlorine ions by calcination under Ar gas at 550 °C [27]. The chlorine ions largely influence the activity of supported metal catalytic materials [31,32,33,34]. The *T*_100_ values of Pd/C-EP and Pd/C-HP were higher than those of Pd/C-EP-Ar and Pd/C-HP-Ar because of the presence of Cl^−^ in the samples. The XPS analysis results of Cl 2p also corresponded to the activity results of the Pd/C catalytic materials on CO oxidation reactivity.

Figure 5c presents the C1s XPS spectra in Pd/C-EP and Pd/C-HP. The binding energy at 284.6 eV for the samples is ascribed to the adventageous carbon or graphite carbon. Compared to Pd/C-HP, there is a new weak peak at ca. 285.5 eV, attributing to the amorphous carbon layer formed by ethanol decomposition during plasma treatment. This phenomenon is also obtained by using AP CO cold plasma to reduce the metal ions supported on TiO_2_ support [35].

As shown in Table 2, the proportions of metal Pd^0^ on the surfaces of Pd/C-HP and Pd/C-EP are 61.4% and 52.1% respectively, and those of Pd/C-HP-Ar and Pd/C-EP-Ar prepared by calcination in Ar gas are 58.7% and 42.6%, respectively. The proportions of Pd^0^ decreased after calcination, considering the oxidation of the Pd nanoparticles by the oxygen-containing functional groups on the support surface at high-temperature calcination. The catalytic activity of a material is related to its Pd content. Pd/C-HP and Pd/C-EP, which contained large amounts of Pd^0^, exhibited significantly lower activity, compared with Pd/C-HP-Ar and Pd/C-EP-Ar, which contained small amounts of Pd^0^, because of the influence of Cl^−^ ions on the materials.

**Figure 6:**
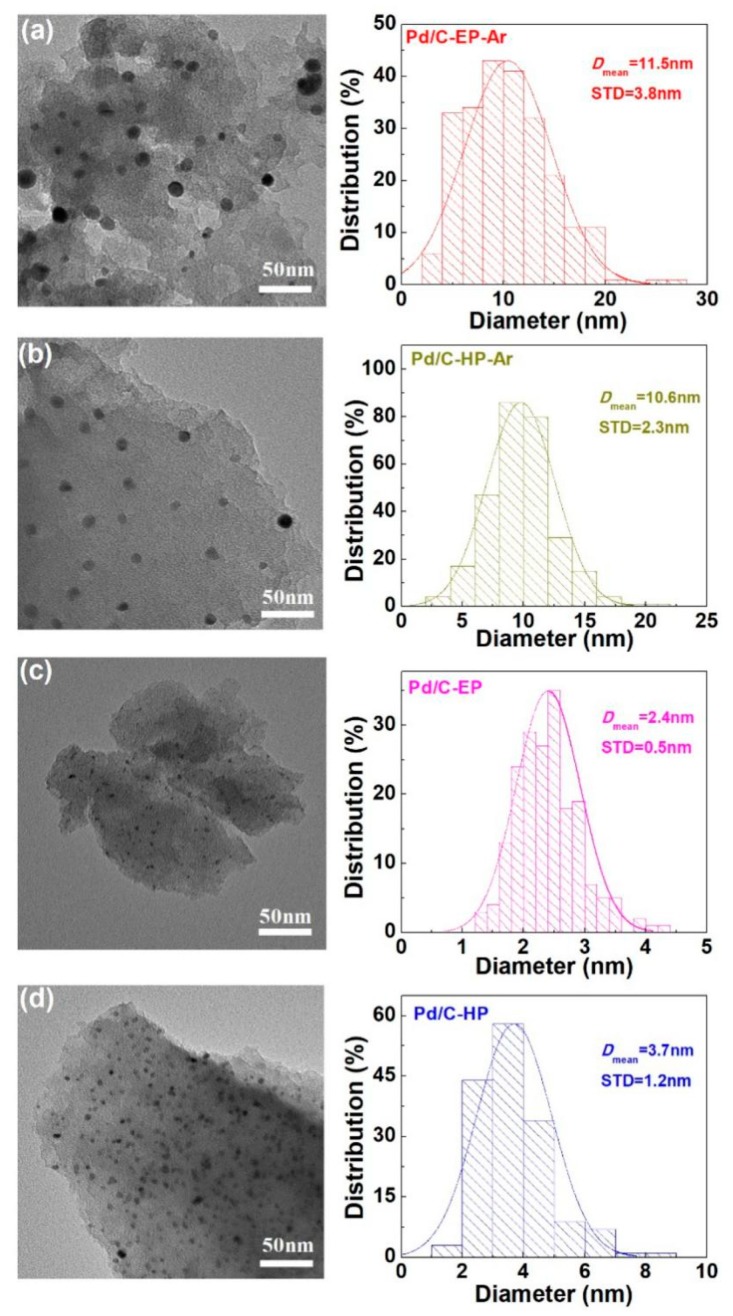
Typical transmission electron microscopy (TEM) images of (**a**) Pd/C-EP-Ar, (**b**) Pd/C-HP-Ar, (**c**) Pd/C-EP, and (**d**) Pd/C-HP and the corresponding histograms of the size distribution of the Pd nanoparticles.

Figure 6 shows the TEM images and the Pd nanoparticle size distribution in the Pd/C catalytic materials. Pd/C-EP, Pd/C-EP-Ar, Pd/C-HP, and Pd/C-HP-Ar had average Pd nanoparticle sizes of 2.4 ± 0.5, 11.5 ± 3.8, 3.7 ± 1.2, and 10.6 ± 2.3 nm respectively, which were consistent with the XRD results. The Pd nanoparticles for Pd/C-EP and Pd/C-HP were smaller and uniformly dispersed on the surface of the support, which conformed to the characteristics of supported metal catalysts prepared using AP cold plasma [25,26,27,29]. Pd/C-EP had the smallest Pd nanoparticles because of the carbon layer formed by ethanol decomposition, which can protect the Pd nanoparticles and inhibit their aggregation. The Pd nanoparticles agglomerated when the materials were calcined at a high temperature, resulting in larger Pd nanoparticles in Pd/C-EP-Ar and Pd/C-HP-Ar. In addition, the sizes and distribution of the Pd nanoparticles in Pd/C-EP-Ar were not uniform because of the destruction of the carbon layer caused by high-temperature calcination and uneven agglomeration of Pd nanoparticles. The results indicated that Pd ions loaded on activated carbon could be reduced using AP ethanol cold plasma. Moreover, the small Pd nanoparticles and uniform distribution in Pd/C-EP is attributed to the carbon layer protection, however, after calcination, Pd/C-EP-Ar had a larger Pd nanoparticle size. In spite of this, Pd/C-EP-Ar had similar average size of Pd nanoparticles with Pd/C-HP-Ar. Most importantly, explosive hydrogen gas is avoided for synthesizing the Pd/C catalytic material.

**Figure 7:**
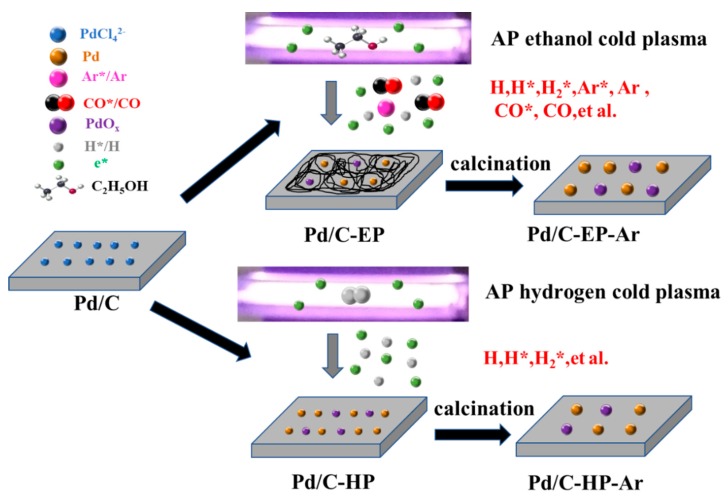
Schematic of the proposed preparation mechanism of Pd/C-EP-Ar and Pd/C-HP-Ar catalytic materials.

The proposed preparation mechanisms of Pd/C-EP-Ar and Pd/C-HP-Ar were determined from the results of the XPS, TEM, and XRD analyses, among others (Figure 7). The interaction of e* with ethanol during AP ethanol cold plasma treatment can induce not only the excited states of the Ar* by the collision of high-energy electrons (e*) with Ar gas but also active hydrogen species (H, H*, H_2_*), both of which can reduce metal ions. Meanwhile, CO and the excited state of CO* produced by ethanol decomposition can also reduce Pd ions, and the carbon layer generated by ethanol decomposition can inhibit the agglomeration of Pd nanoparticles. Thus, Pd/C-EP with the smallest Pd nanoparticles size was fabricated [24,25,35]. However, Pd/C-EP-Ar with large Pd nanoparticles was produced after high-temperature calcination in Ar gas to remove chlorine ions. Pd ions were reduced by the active hydrogen species (H, H*, H_2_*) in the preparation of AP hydrogen cold plasma. They are produced by high-energy electrons e* colliding with Ar and H_2_ gases. Pd/C-HP-Ar with larger Pd nanoparticles can be formed when Pd/C-HP is calcined in Ar gas to remove chloride ions.

In summary, both AP ethanol cold plasma and AP hydrogen cold plasma can reduce the Pd ions into metallic Pd nanoparticles. The Pd/C-HP and Pd/C-EP catalytic materials exhibited significantly lower CO oxidation activity in spite of their large amounts of metallic Pd^0^. The low activity of Pd/C-HP is attributed to the influence of Cl^−^ ions, while it is ascribed to the influence of Cl^−^ ions and the protection of the carbon layer formed by ethanol decomposition in plasma for Pd/C-EP. In addition, the Pd/C atomic ratio for Pd/C-EP (0.013) is lower than that for Pd/C-HP (0.026) due to the carbon layer protection. Therefore, Pd/C-EP exhibits lower CO oxidation reactivity than Pd/C-HP. In contrast, both Pd/C-HP-Ar and Pd/C-EP-Ar exhibit high CO oxidation activity notwithstanding the large size of Pd nanoparticles due to the high-temperature calcination. Compared to the Pd/C-HP and Pd/C-EP catalytic materials, the enhanced performance of the Pd/C-HP-Ar and Pd/C-EP-Ar samples is mainly attributed to the removal of Cl^−^ ions. In addition, the average size of Pd nanoparticles in Pd/C-HP-Ar is smaller than that in Pd/C-EP-Ar, while the Pd/C atomic ratio in Pd/C-EP-Ar is higher than that in Pd/C-HP-Ar due to the protection of the carbon layer. Therefore, both of them exhibit high CO oxidation activity.

## 4. Conclusions

Pd/C catalytic materials were prepared by treatment with AP ethanol cold plasma and AP hydrogen cold plasma. The structure and properties of the materials were characterized, and the preparation mechanism was investigated. The results of TEM, XPS, and XRD analyses show that both methods can reduce the Pd ions loaded on the activated carbon to the metal state. Not only the active hydrogen species but also reductive species, such as active CO* species, can be formed during treatment with AP ethanol cold plasma. All of these species, produced by ethanol decomposition, can reduce the Pd ions. The Pd nanoparticles in Pd/C-EP were smaller than those in Pd/C-HP because of the carbon layer formed via ethanol decomposition, which effectively prevented the agglomeration of Pd nanoparticles. The results of CO oxidation reaction indicated that the 100% CO conversion temperature (*T*_100_) of Pd/C-EP-Ar is 140 °C, which was similar to that of Pd/C-HP-Ar prepared using AP hydrogen cold plasma. AP ethanol cold plasma and AP hydrogen plasma can be effectively used to fabricate Pd/C catalytic materials. The use of H_2_, which is explosive and difficult to transport, was avoided in the treatment with AP ethanol cold plasma. This study carries considerable research significance and application potential for the fabrication of supported metal catalytic materials.

## Figures and Tables

**Table 1:** Specific surface area, pore diameter, and pore volume of the Pd/C catalytic materials.

Samples	Pore Diameter (nm)	Pore Volume (cm^3^·g^−1^)	Specific Surface Area (m^2^·g^−1^)
Pd/C-EP-Ar	3.82	0.50	874
Pd/C-HP-Ar	3.82	0.47	871
Pd/C-EP	3.83	0.41	703
Pd/C-HP	3.83	0.41	736
Pd/C	3.84	0.39	690

**Table 2:** Pd composition and Pd/C atomic ratios in the Pd/C catalytic materials.

Catalyst	Pd Composition (%)			Pd/C Atomic Ratio
	Pd^0^	Pd^2+^	Pd^4+^	
Pd/C-EP-Ar	42.6	36.2	21.2	0.023
Pd/C-HP-Ar	58.7	19.8	21.5	0.010
Pd/C-EP	52.1	21.4	26.5	0.013
Pd/C-HP	61.4	31.1	7.5	0.026
